# JournalTOCs

**DOI:** 10.5195/jmla.2018.452

**Published:** 2018-07-01

**Authors:** Rob Penfold

**Affiliations:** Barwon Health Library, Barwon Health, Geelong, Victoria, Australia

Many clinical libraries offer a journal alerting service that gives clinicians the opportunity to review the most current tables of contents for journals of interest. Benefits of this service include helping clinicians keep up to date with their specific areas of clinical practice or research; increasing usage and awareness of expensive journal subscriptions; cross-promoting other library resources, services, and news; regularly reminding clinicians that the library exists and is available to provide other services; and improving customer satisfaction [1–3].

Mechanisms for providing a journal alerting service have evolved over the years, moving from simple paper-based approaches to electronic tables of contents (e-TOCs) services. As the number of potential e-TOCs increases, it becomes increasingly unwieldly to register and sign up for alerts with multiple publishers. JournalTOCs circumvents this issue by aggregating e-TOCs so that they are available from one online resource.

JournalTOCs is a portal provided by Heriot-Watt University [4, 5]. Alerts are available for 29,882 journals from 2,948 publishers and cover 73 broad subject areas (which in turn break down into multiple smaller subject areas). While JournalTOCs is built on really simple syndication (RSS) (and so is limited to journals that support that protocol), the e-TOCs are delivered to end users as email alerts.

The free version of JournalTOCs allows users to sign up for 30 journal alerts. This review, however, focuses on JournalTOCs Premium, which allows clinical libraries to provide a comprehensive email journal alerting service for clients, with features such as 300 journal alerts per user, customized library messages in alert emails, and integration with an institution’s journal subscriptions for full-text access. Given this use case, the experience with JournalTOCs Premium will be described from the perspective of both the end user and the institution.

## THE USER EXPERIENCE

Take a random client (let’s call him Stu Dent). He has received a message from his library that they have subscribed to JournalTOCs Premium. Stu registers with his institutional email address, as this makes his institution’s subscribed journal collection available to him. Stu can find journals of interest by browsing subjects, browsing publishers, or searching and then selecting journals to “follow” (i.e., for which to receive the tables of contents).

On the JournalTOCs Home page, he can see his journals and their access levels: a green square for journals that his institution subscribes to, a green OA for open access journals, a divided green/red box for journals having some open access articles, and a lock icon for journals that are not directly accessible to him. Stu will now start receiving emails with new journal content and can update his selections at any time.

## THE INSTITUTION EXPERIENCE

When an institution subscribes to JournalTOCs Premium, the first step is to upload a list of its subscribed journals. The list must be in .csv format with a maximum of 2,000 records per upload, so multiple uploads may be required.

The Service Configuration section has numerous settings, organized by category. The Look and Feel section settings include welcome text, library email and website details, and a logo that clients will see on the JournalTOCs home page. A reference manager format (Endnote, Zotero, etc.) can be specified here. In the Full Text Access section, access can be configured by OpenURL, EZProxy, and Internet protocol (IP) range. There are also options for allowing proxied uniform resource locators (URLs) in alerts and including a Request a Copy link in emails. The Email Alert section allows users to specify that alerts are sent journal by journal (rather than as a digest), with various additional options relating to frequency, link structure, and email appearance. The Email Templates section enables customization of various emails (registration, e-TOCs, etc.), including header and footer images (Logos tab) and text (including HTML links). Templates are easy to update and provide a very convenient channel for alerting clients to new library resources and services.

JournalTOCs Premium allows a variety of e-TOC alert options, which can be patron driven, library driven, or somewhere in between. In the latter option for example, library staff could add or remove e-TOC subscriptions for an existing client account. This feature allows flexibility, as not all clients are comfortable setting up alerts themselves. An additional example of a hybrid approach is that library staff can set up a single journal alert for all new staff, after which users can either add or remove alerts depending on their preferences.

JournalTOCs Premium offers an application programming interface (API) consisting of four calls (journals, articles, user, and institution). Some sites have used the API to enable e-TOC subscriptions from their own discovery systems. Note that this integration requires liaison with the vendor.

Usage Reports are available for metrics that include journals followed and clients and their e-TOC subscriptions. Figures for article titles clicked (as a proxy for full-text articles for subscribed content) or Request a Copy links clicked are not available, unfortunately; these would be very useful in evaluating impact.

JournalTOCs is currently in the process of adding a Request Article link at the article level for all alerts, which will strengthen its value to clients and subscribing libraries.

## JOURNALTOCS PREMIUM VERSUS ALERTING APPS

A number of apps provide alerts for clinical content, including BrowZine [6], Read [7], and Docphin [8, 9]. Inspection of the Google Play Store in January 2018 revealed approximately 5,000 downloads for the Docphin app, approximately 10,000 for the BrowZine app, and approximately 100,000 for the Read app, so the latter seemed to be the clear leader in this space. It is beyond the scope of this short review to compare all features, strengths, and weaknesses of these apps, but it does address reasons why a library might consider a service like JournalTOCs Premium instead of or in addition to these apps. All allow access to subscribed content, so that is not a differentiating factor. The particular strengths of JournalTOCs can be identified in four main areas.

### New journal issue alerts

JournalTOCS is probably the closest to a traditional new journal issue alert. Technically, BrowZine is not an alerting service at all; clients must remember to return to the app or site to view new content. This reviewer has had the experience of signing up for similar services only to forget about them completely and then one day return to the services to find thousands of new items. An alerting capability is on the BrowZine development roadmap [10], and if implemented, this feature will enhance its value. Docphin only provides keyword alerts, while Read has a journal alert but seems to only give a few articles from each issue. While keyword alerts have their place, whole journal issue alerts expose a wider range of content.

### Library promotion

Perhaps the most powerful advantage of JournalTOCs is the ability to deliver customized messages and links regularly into clients’ inboxes. Libraries can easily update this messaging feature to cross-promote other library resources and services. More generally, the steady flow of emails to clients serves as a reminder that the library exists and is available to assist in other areas besides journal alerts.

### Web access

JournalTOCs, BrowZine, Read, and Docphin all have browser-based interfaces. The latter three also have apps, and this is frequently the channel by which they are promoted. In some ways, it seems curious to suggest that only having a browser version is an advantage. However, browsers accessed via a computer and email are near universal for health services staff, so there is less of a technology barrier than with mobile alternatives.

### Pricing

This review has explored JournalTOCs Premium, which is a subscription product. The website specifies, “The price of an annual licence starts at £480.00 GBP and is in proportion to the number of journals, the estimated number of user accounts, the amount of tailored unique features requested for the customization and the type of your organisation (e.g. non-for-profit institutions)” [11]. This would seem to be broadly comparable with competitor pricing.

## CONCLUSION

With the ever-growing amount of information, clients can often be overwhelmed when their searches return very large numbers of search results, whether via a web search engine or the library’s discovery system. In contrast, journal content alerts provide a neatly packaged set of results with a manageable number of items focused on a journal’s specific subject area. Several vendors seem to recognize this advantage and have products existing or in development that will compete with JournalTOCs. A noteworthy example is Ovid, which is in the process of introducing its discovery product (Ovid Discovery), which includes e-TOCs for approximately 16,000 journals via its Journals listing interface. Not surprisingly, the journal alerts space has matured over time. Libraries now have a number of sophisticated options available to them to help their clients stay up to date and, in the process, help promote the library service.
